# The patient journey and burden of disease in progressive pulmonary fibrosis in Japan: a cross-sectional survey

**DOI:** 10.3389/fmed.2025.1526530

**Published:** 2025-04-11

**Authors:** Yuko Kaneko, Yuko Waseda, Hidekata Yasuoka, Masateru Okazaki, Shoko Nagata, Ryoko Iwasaki, Mark Small, Haruyuki Ishii

**Affiliations:** ^1^Division of Rheumatology, Department of Internal Medicine, School of Medicine, Keio University, Tokyo, Japan; ^2^Division of Respiratory Medicine, University of Fukui Hospital, Eiheiji, Japan; ^3^Division of Rheumatology, Department of Internal Medicine, School of Medicine, Fujita Health University, Toyoake, Japan; ^4^Nippon Boehringer Ingelheim Co., Ltd, Tokyo, Japan; ^5^Adelphi Real World, Bollington, United Kingdom; ^6^Department of Respiratory Medicine, Faculty of Medicine, Kyorin University, Tokyo, Japan

**Keywords:** idiopathic pulmonary fibrosis, progressive pulmonary fibrosis, interstitial lung disease, antifibrotics, treatment, real-world data, health-related quality of life

## Abstract

**Background:**

For patients with interstitial lung diseases (ILDs) other than idiopathic pulmonary fibrosis (IPF) with a progressive pulmonary fibrosis (PPF) phenotype, current knowledge of patient experience and symptom burden is limited. This study aimed to describe the patient journey for patients with PPF and IPF in a real-world setting in Japan.

**Methods:**

Data were analyzed from the Adelphi Real World PPF-ILD Disease Specific Programme™, a cross-sectional survey with elements of retrospective data collection of pulmonologists and rheumatologists in Japan from April to October 2022. Participants provided data for up to 12 consecutive patients with physician-confirmed ILD with a progressive phenotype. Analyses were descriptive, except Kappa (κ) statistic was used to measure the alignment between physician- and patient-reported symptom burden in the 4 weeks prior to survey date (poor agreement: κ =<0.00; slight 0.00–0.20; fair 0.21–0.40; moderate 0.41–0.60; substantial 0.61–0.80; almost perfect 1.00).

**Results:**

A total of 63 physicians (43 pulmonologists and 20 rheumatologists) provided data on 382 patients (312 with PPF and 70 with IPF). These patients were also asked to complete a voluntary survey on their experience and symptoms. Mean time from first symptom to consultation was 14.1 months for IPF, 8.0 months for non-connective tissue disease (CTD)-associated ILDs, and 10.7 months for CTD-ILDs. Mean times from consultation to diagnosis were 7.1, 4.8, and 3.6 months, respectively. Perception of symptoms differed between physicians and patients with alignment ranging from poor (dysphagia, κ = –0.0296, *p* = 0.6217) to substantial (weight loss, κ = 0.6174, *p* = 0.001). Health-related quality of life (HRQoL) was consistently impaired in patients overall, but too few patients completed HRQoL instruments to compare IPF with other forms of ILD.

**Conclusions:**

This real-world study expands our understanding of the patient journey for patients with PPF and IPF in Japan. Greater communication between patients and physicians is needed to shorten diagnostic delays and target treatment strategies to improve patient experience and overall outcomes.

## 1 Introduction

Idiopathic pulmonary fibrosis (IPF) is an interstitial lung disease (ILD) characterized by chronic progressive fibrosis, worsening of lung function and dyspnoea (1–3). While IPF is always progressive, ILD may also be associated with other underlying diseases, in response to external irritants, or from other causes (4, 5). The extent to which other ILDs are fibrosing is variable, and chronic fibrosing ILDs other than IPF may or may not show a progressive phenotype (4, 5). Those that are progressive can be grouped together under the term “progressive pulmonary fibrosis” (PPF) (1).

Treatment of PPF is based on treatments for the underlying disease along with antifibrotic therapy shown to be effective in IPF. However, while the clinical course of PPF may be similar to IPF in some patients, rates of progression and other aspects of disease may vary markedly among others (4, 5). It is clear, therefore, that findings and conclusions drawn from studies in IPF cannot necessarily be extrapolated to patients with PPF.

The burden of IPF to patients and the patient journey during diagnosis and treatment has been widely investigated (6–10), with patients consistently reporting delays in diagnosis, misdiagnosis, delays in treatment and negative effects on health-related quality of life (HRQoL). In PPF, the burden and journey for patients have also been investigated, though to a lesser extent (11–13). A fuller understanding of the burden of IPF and PPF for patients and of their treatment journey is important to ensure optimal treatment strategies and resource allocation.

To understand the patient burden and journey of PPF in Japan, and compare it with IPF, we have conducted an analysis of data from a cross-sectional survey of both physicians, and patients with PPF and IPF.

## 2 Materials and methods

### 2.1 Data source

Data were drawn from the Adelphi Real World PPF-ILD Disease Specific Programme™ (DSP), a large, real-world, cross-sectional survey with elements of retrospective data collection of ILD patients. The DSP was conducted in Japan from April to September 2022. The DSP methodology has been previously described, validated and found to be representative and consistent over time (14–16). Physicians reported data on patient demographics, clinical characteristics, symptom burden and impact, patient management, treatment utilization, and decision-making in routine care. This study analyzed data from the Japanese population within the original cohort. Target physicians in Japan (pulmonologists and rheumatologists) were identified from public lists of healthcare professionals (HCPs). To be included in the study, pulmonologists were required to see at least four different types of qualifying ILDs in a typical month, and rheumatologists were required to see at least two different types of connective tissue disease (CTD)-associated ILD (rheumatoid arthritis-associated ILD, systemic sclerosis-associated ILD, polymyositis/dermatomyositis-ILD or Sjögren’s-associated ILD) in a typical month. Participating physicians completed surveys, which provided general information on management, referrals, usage, and awareness of antifibrotics and attitudes toward PPF, as well as patient record forms (PRFs) for up to 12 consecutively consulted patients. Patients were eligible for inclusion if they were aged over 18 years, had a physician-confirmed diagnosis of ILD and presented with a progressive phenotype (PPF), as determined by the reporting physician (which could include IPF). The clinical characteristics and treatment profile of this patient cohort are described in a separate manuscript (17), whereas the patient journey and burden of IPF and PPF from both the physician and patient perspective are reported here.

Physician- and patient-reported disease severity were categorized as mild, moderate or severe, and progression status as improving, stable or progressing. In each case, the categories were based on physician or patient opinion and not predefined. The study was performed prior to publication of the current American Thoracic Society/European Respiratory Society/Japanese Respiratory Society/Asociación Latinoamericana de Tórax guidelines (1), and no clinical definition of a progressive phenotype was prespecified.

Physicians provided information on patient demographics, the patient’s ILD journey and symptom burden as part of the PRF. Patients with a completed PRF were also asked by their physician to complete a patient self-report form (PSC) immediately after consultation, although this was entirely voluntary.

Patients were also asked to complete the Work Productivity and Impairment Questionnaire: Specific Health problems (WPAI-SHP), consisting of six questions addressing employment status, hours worked, work time missed due to health, work time missed for other reasons, effects on productivity in work, and effects on productivity outside work (18); the EuroQol EQ5D utility and EQ5D-5L visual analogue scale (19), and the Kings’ Brief Interstitial Lung Disease (KBILD) instrument, an ILD-specific instrument to measure the effect of ILD on patients’ lives (20). The EQ5D-5L score was calculated for patients who completed all parts of the EQ5D utility. This is a composite score derived from patient responses on all of the EQ5D dimensions data, ranging from 0.00 as the worst imaginable health state to 1.00 as the best imaginable health state.

### 2.2 Data analyses

Patients were grouped according to type of ILD, into those with IPF, those with ILDs due to underlying CTDs, and ILDs with other underlying causes. The CTD-ILD group included patients with ILD associated with rheumatoid arthritis, systemic sclerosis, dermatomyositis/polymyositis or Sjögren’s while the non-CTD-ILD group included patients with idiopathic non-specific interstitial pneumonia, chronic hypersensitivity pneumonitis and unclassifiable ILD.

As the primary research objective was descriptive in nature (i.e., no *a priori* hypotheses specified), the available sample size of physicians and patients was driven by the DSP data collection methodology. Therefore, formal sample size calculations were not applicable and were not performed and the sample size impacted the precision of any estimates. Descriptive analyses were undertaken by Adelphi Real World and conducted in UNICOM^®^ Data Collection Survey Reporter (UNICOM^®^ Global, Inc, Mission Hills, CA, United States).

For age-based patient milestones (e.g., age at first HCP visit, age at first symptom, age at diagnosis), only patients with a value recorded for each of the milestones were included. A Kappa (κ) statistic was performed to measure the alignment between physician- and patient-reported symptom burden in the 4 weeks prior to survey date (poor agreement κ < 0.00; slight κ = 0.00–0.20; fair κ = 0.21–0.40; moderate κ = 0.41–0.60, substantial κ = 0.61–0.80; almost perfect κ = 1.00). Physicians and patients reported the presence of symptoms from a pre-coded list via a checkbox. Symptoms captured within the physician and patient pre-coded list of symptoms were included in the analysis. Because fatigue and tachycardia were included in the physician symptom list but not the patient list they were excluded from the analysis. For the analysis, the categories of chest pain and chest pressure/tightness in the physician symptom list were combined to align with chest pain/tightness in the patient symptom list.

### 2.3 Ethics statement

Data were collected by local fieldwork partners, and both physician and patient data were de-identified prior to receipt by Adelphi. The DSP has received Pearl Institutional Review Board ethical exemption (exemption code 22-ADRW-135) and was conducted adhering to European Pharmaceutical Market Research Association guidelines of which Japan is a signatory.

## 3 Results

### 3.1 Participants

A total of 63 physicians (43 pulmonologists and 20 rheumatologists) completed PRFs for 382 patients (312 with PPF and 70 with IPF), while 68 patients completed PSCs (Supplementary Table 1). Baseline patient characteristics are described elsewhere (17). The mean age was 71.9 years for patients with IPF [± standard deviation (SD) 7.3 years], 64.1 (± 12.6) years for patients with CTD-ILD, and 70.6 (± 10.8) years for patients with other ILDs. Among patients with IPF, 12.9% were female, while 65.3% of patients with CTD-ILD and 33.1% of patients with other ILDs were female.

### 3.2 Time to diagnosis

As reported by physicians, 214/245 patients with data (87.3%) had experienced symptoms before diagnosis whereas 40/66 patients who completed the relevant part of the PSC form (60.6%) reported that they had experienced symptoms concerning their lungs or breathing conditions before diagnosis. The most common symptoms reported prior to diagnosis were dyspnoea/breathlessness on exertion (physician-reported 65.4%, patient-reported 55.0%), cough (physician-reported 55.1%, patient-reported 70.0%), chest pressure/tightness (physician-reported 19.6%) chest pain/tightness (patient-reported 15.0%) and dyspnoea/breathlessness following exertion (physician-reported 15.4%, patient-reported 45.0%), while other symptoms unrelated to breathing conditions were also reported (Table 1). The mean age at which patients first experienced symptoms was 68.5 ± 8.06 years for patients with IPF, 66.9 ± 11.11 years for patients with non-CTD ILDs, and 61.7 ± 12.93 years for patients with CTD-ILDs.

**TABLE 1:** Signs and symptoms reported prior to diagnosis.

	All patients	IPF	Non-CTD-ILDs	CTD-ILDs
**Physician-reported signs and symptoms prior to diagnosis, *n* (%)**	***n*** = 245	***n*** = 55	***n*** = 101	***n* = 89**
Yes	214 (87.3)	52 (94.5)	92 (91.1)	70 (78.7)
No	31 (12.7)	3 (5.5)	9 (8.9)	19 (21.3)
**Physician-reported signs and symptoms, *n* (%)**	***n* = 214**	***n* = 52**	***n* = 92**	***n* = 70**
Dyspnoea on exertion	140 (65.4)	36 (69.2)	52 (56.5)	52 (74.3)
Cough	118 (55.1)	28 (53.9)	55 (59.8)	35 (50.0)
Chest pressure/tightness	42 (19.6)	10 (19.2)	15 (16.3)	17 (24.3)
Dyspnoea following exertion	33 (15.4)	9 (17.3)	12 (13.0)	12 (17.1)
Velcro crackles	32 (15.0)	6 (11.5)	13 (14.1)	13 (18.6)
Dyspnoea at rest	23 (10.8)	4 (7.7)	11 (12.0)	8 (11.4)
Fatigue	23 (10.8)	4 (7.7)	6 (6.5)	13 (18.6)
Clubbed fingers	18 (8.4)	7 (13.5)	7 (7.6)	4 (5.7)
Chest pain	12 (5.6)	3 (5.8)	4 (4.4)	5 (7.1)
Weight loss	11 (5.1)	1 (1.9)	1 (1.1)	9 (12.9)
Reduced exercise tolerance	7 (3.3)	2 (3.9)	3 (3.3)	2 (2.9)
Wheezing	7 (3.3)	2 (3.9)	2 (2.2)	3 (4.3)
Decreased appetite	6 (2.8)	1 (1.9)	3 (3.3)	2 (2.9)
Dyspnoea when exposed to trigger	3 (1.4)	2 (3.9)	0 (0)	1 (1.4)
Other	3 (1.4)	0 (0)	2 (2.2)	1 (1.4)
Tachycardia	2 (0.9)	0 (0)	1 (1.1)	1 (1.4)
Dizziness	1 (0.5)	0 (0)	0 (0)	1 (1.4)
Dysphagia	1 (0.5)	0 (0)	0 (0)	1 (1.4)
Haemoptysis	1 (0.5)	0 (0)	1 (1.1)	0 (0)
**Patient-reported symptoms prior to diagnosis**	***n* = 66**	***n* = 9**	***n* = 31**	***n* = 26**
Yes	40 (60.6)	7 (77.8)	19 (61.3)	14 (53.9)
No	26 (39.4)	2 (22.2)	12 (38.7)	12 (46.2)
**Patient-reported symptoms, *n* (%)**	***n* = 40**	***n* = 7**	***n* = 19**	***n* = 14**
Chest pain/tightness	6 (15.0)	0 (0.0)	3 (15.8)	3 (21.4)
Cough	28 (70.0)	5 (71.4)	15 (79.0)	8 (57.1)
Swollen/rounded fingers	3 (7.5)	0 (0.0)	0 (0.0)	3 (21.4)
Decreased appetite	4 (10.0)	0 (0.0)	2 (10.5)	2 (14.3)
Dizziness	2 (5.0)	1 (14.3)	0 (0.0)	1 (7.1)
Breathlessness following exertion	18 (45.0)	4 (57.1)	7 (36.8)	7 (50.0)
Breathlessness on exertion	21 (55.0)	4 (57.1)	10 (52.6)	8 (57.1)
Breathlessness at rest	1 (2.5)	0 (0.0)	1 (5.3)	0 (0.0)
Breathlessness when exposed to a trigger (e.g., dust, strong odors)	3 (7.5)	1 (14.3)	1 (5.3)	1 (7.1)
Heart burn	2 (5.0)	0 (0.0)	0 (0.0)	2 (14.3)
Fatigue	8 (20.0)	1 (14.3)	2 (10.5)	5 (35.7)
Irregular heartbeat	2 (5.0)	1 (14.3)	0 (0.0)	1 (7.1)
Wheezing	3 (7.5)	2 (28.6)	0 (0.0)	1 (7.1)
Weight loss	4 (10.0)	0 (0.0)	1 (5.3)	3 (21.4)
Other	2 (5.0)	0 (0.0)	1 (5.3)	1 (7.1)
Unknown	2 (5.0)	0 (0.0)	0 (0.0)	2 (14.3)

CTD, connective tissue disease; ILD, interstitial lung disease; IPF, idiopathic pulmonary fibrosis.

**FIGURE 1:**
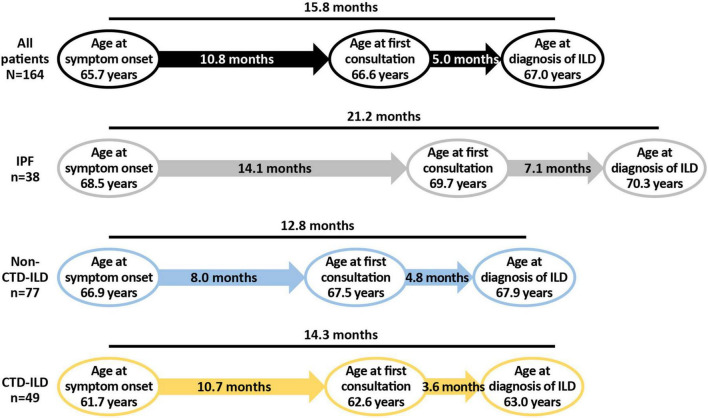
Mean time to first consultation for ILD and time to diagnosis. CTD, connective tissue disease; ILD, interstitial lung disease; IPF, idiopathic pulmonary fibrosis.

Patients with IPF reported a mean time from first symptoms to their first consultation for their symptoms of 14.1 ± 21.08 months. The mean time for those with non-CTD-ILDs was 8.0 ± 10.72 months and for those with CTD-ILDs, it was 10.7 ± 30.69 months. Following consultation, the mean time to diagnosis of ILD was 7.1 ± 18.17 months for patients with IPF, 4.8 ± 7.67 months for those with non-CTD-ILDs, and 3.6 ± 10.5 months for those with CTD-ILDs (Figure 1).

### 3.3 Initial consultation and referral

As reported by physicians, the first consultation for ILD was with a primary care physician for 36.4% of patients overall, 40.0% of those with IPF, 37.2% with non-CTD-ILDs, and 34.1% with CTD-ILDs. Around half of patients with IPF (48.6%) and non-CTD-ILDs (52.4%) were first seen by a pulmonologist/respiratory specialist, compared with 24.0% of those with CTD-ILDs. Of patients with CTD-ILDs, 30.5% were first seen by a rheumatologist (Table 2).

**TABLE 2:** First consultation for ILD (physician-reported).

	All patients	IPF	Non-CTD-ILDs	CTD-ILDs
**Physician first seen regarding ILD, *n* (%)**	***N* = 382**	***n* = 70**	***n* = 145**	***n* = 167**
Pulmonologist/respiratory specialist	150 (39.3)	34 (48.6)	76 (52.4)	40 (24.0)
Primary care physician	139 (36.4)	28 (40.0)	54 (37.2)	57 (34.1)
Rheumatologist	52 (13.6)	1 (1.4)	0 (0)	51 (30.5)
Internist	18 (4.7)	4 (5.7)	6 (4.1)	8 (4.8)
Dermatologist	7 (1.8)	0 (0)	0 (0)	7 (4.2)
Other HCP	7 (1.8)	2 (2.9)	4 (2.8)	1 (0.6)
Cardiologist	5 (1.3)	1 (1.4)	2 (1.4)	2 (1.2)
Emergency department physician	3 (0.8)	0 (0)	3 (2.1)	0 (0)
Respiratory nurse	1 (0.3)	0 (0)	0 (0)	1 (0.6)
Internal medicine	0 (0)	0 (0)	0 (0)	0 (0)

CTD, connective tissue disease; HCP, healthcare professional; ILD, interstitial lung disease; IPF, idiopathic pulmonary fibrosis.

Just over half of patients with IPF (38/70, 54.3%) had been referred to their current pulmonologist/rheumatologist by another healthcare professional. The figure was higher for patients with non-CTD-ILDs (85/145, 58.6%) and CTD-ILDs (110/167, 65.9%). Referral to the treating pulmonologist/rheumatologist was by a primary care physician for almost two-thirds of patients referred with IPF (65.8%), and a similar percentage of patients with non-CTD-ILDs (56.5%), whereas only 35.5% of patients with CTD-ILDs had been referred by a primary care physician (Table 3). The most reported reasons for referral were not specializing/lack of knowledge in respiratory conditions (40.0%–67.1%) (Table 3). Diagnosis of ILD was carried out by a pulmonologist in almost all patients with IPF (95.7%) and non-CTD-ILDs (99.3%) but in only 28.1% of those with CTD-ILDs, where a rheumatologist made the diagnosis in 70.1% of patients (Table 4).

**TABLE 3:** Referring HCPs and reasons for referral.

	All patients	IPF	Non-CTD-ILDs	CTD-ILDs
**Person who referred patient**	***N* = 233**	***n* = 38**	***n* = 85**	***n* = 110**
Primary care physician	112 (48.1)	25 (65.8)	48 (56.5)	39 (35.5)
Pulmonologist/respiratory specialist	52 (22.3)	3 (7.9)	24 (28.2)	25 (22.7)
Dermatologist	97 (3.0)	0 (0)	0 (0)	7 (6.4)
Internist	21 (9.0)	4 (10.5)	3 (3.5)	14 (12.7)
Rheumatologist	22 (9.4)	0 (0)	0 (0)	22 (20.0)
Cardiologist	6 (2.6)	2 (5.3)	2 (2.4)	2 (1.8)
Other HCP	8 (3.4)	3 (7.9)	4 (4.7)	1 (0.9)
Emergency department physician	6 (2.2)	1 (2.6)	4 (4.7)	0 (0)
**Reason for referral**	***N* = 233**	**38**	**85**	**110**
Referring HCP is not specialized in respiratory conditions	125 (53.7)	24 (63.2)	57 (67.1)	44 (40.0)
Referring HCP lack of understanding around ILD	57 (24.5)	11 (29.0)	22 (25.9)	24 (21.8)
Additional diagnostic testing required (e.g., HRCT, blood tests, spirometry)	41 (17.6)	8 (21.1)	13 (15.3)	20 (18.2)
Patient referred to treat the underlying disease	18 (7.7)	0 (0)	0 (0)	18 (16.4)
Uncertainty regarding disease prognosis	17 (7.3)	2 (5.3)	3 (3.5)	12 (10.9)
Patient requested referral	15 (6.4)	1 (2.6)	8 (9.4)	6 (5.5)
Patient has complications that I am best placed to manage	12 (5.2)	0 (0)	2 (2.4)	10 (9.1)
Patient has other conditions that I am best placed to manage	7 (3.0)	0 (0)	0 (0)	7 (6.4)
Referring HCP has exhausted all therapy options available to them	5 (2.2)	1 (2.6)	1 (1.2)	3 (2.7)
Patient considered for antifibrotic treatment and the referring HCP is not permitted/comfortable prescribing them	4 (1.7)	1 (2.6)	2 (2.4)	1 (0.9)
Additional education required on complications of therapy, problems with adherence, or the management of the disease	3 (1.3)	1 (2.6)	1 (1.2)	1 (0.9)
Other reason	6 (2.6)	2 (5.3)	1 (1.2)	3 (2.7)

CTD, connective tissue disease; HCP, healthcare professional; HRCT, high-resolution computed tomography; ILD, interstitial lung disease; IPF, idiopathic pulmonary fibrosis.

**TABLE 4:** Healthcare professional who diagnosed ILD (physician-reported).

	All patients	IPF	Non-CTD-ILDs	CTD-ILDs
**Physician responsible for diagnosing ILD, *n* (%)**	***N* = 382**	***n* = 70**	***n* = 145**	***n* = 167**
Pulmonologist/respiratory specialist	258 (67.5)	67 (95.7)	144 (99.3)	47 (28.1)
Rheumatologist	118 (30.9)	1 (1.4)	0 (0)	117 (70.1)
Primary care physician	6 (1.6)	2 (2.9)	1 (0.7)	3 (1.8)
**Physician responsible for initiating first treatment regimen for ILD, *n* (%)**	***N* = 321**	***n* = 62**	***n* = 116**	***n* = 143**
Pulmonologist/respiratory specialist	216 (67.3)	60 (96.8)	114 (98.3)	42 (29.4)
Rheumatologist	101 (31.5)	1 (1.6)	0 (0)	100 (69.9)
Primary care physician	4 (1.2)	1 (1.6)	2 (1.7)	1 (0.7)

CTD, connective tissue disease; ILD, interstitial lung disease; IPF, idiopathic pulmonary fibrosis.

A respiratory specialist was responsible for initiating treatment for ILD in almost all patients with IPF and non-CTD-ILDs, whereas in patients with CTD-ILDs a respiratory specialist initiated treatment in less than one-third of patients, with a rheumatologist initiating treatment in 69.9% (Table 4).

Considering data reported by patients who completed the relevant part of the PSC (*n* = 50), findings related to initial diagnosis and referral were comparable with what was reported by physicians on the PRF. Approximately 29.2%–33.3% reported being first seen by a primary care physician and 62.5%–66.7% by a respiratory specialist. The majority of patients (100% IPF, 91.7% non-CTD-ILDs and 82.4% CTD-ILDs) reported that their ILD was diagnosed by a respiratory specialist, although 35.3% of those with CTD-ILD also stated that a rheumatologist diagnosed their ILD (Table 5).

**TABLE 5:** Initial consultation and diagnosis (patient-reported).

	All patients	IPF	Non-CTD-ILDs	CTD-ILDs
**Type of doctor first seen, *n* (%)**	***N* = 50**	***n* = 9**	***n* = 24**	***n* = 17**
Pulmonologist/respiratory specialist	32 (64.0)	6 (66.7)	15 (62.5)	11 (64.7)
Primary care practitioner	15 (30.0)	3 (33.3)	7 (29.2)	5 (29.4)
Other	3 (6.0)	1 (11.1)	2 (8.3)	0 (0)
Rheumatologist	2 (4.0)	0 (0)	0 (0)	2 (11.8)
**Type of doctor who diagnosed lung/breathing condition, *n* (%)**				
Pulmonologist/respiratory specialist	45 (90.0)	9 (100)	22 (91.7)	14 (82.4)
Rheumatologist	6 (12.0)	0 (0)	0 (0)	6 (35.3)
Cardiologist	2 (4.0)	0 (0)	2 (8.3)	0 (0)
Primary care physician	1 (2.0)	0 (0)	0 (0)	1 (5.9)

CTD, connective tissue disease; ILD, interstitial lung disease; IPF, idiopathic pulmonary fibrosis.

### 3.4 Misdiagnosis

Physicians reported that only 6.8% of patients (*n* = 26; 3 with IPF, 11 with non-CTD-ILDs, and 12 with CTD-ILDs) were misdiagnosed with another condition to explain their symptoms before a diagnosis of pulmonary fibrosis was made. The most common conditions that were investigated were asthma (*n* = 6) and chronic obstructive pulmonary disease (*n* = 4) (Table 6).

**TABLE 6:** Misdiagnosis (physician-reported).

	All patients	IPF	Non-CTD-ILDs	CTD-ILDs
**Other conditions diagnosed to explain symptoms? *n* (%)**	***N* = 382**	***n* = 70**	***n* = 145**	***n* = 167**
Yes	26 (6.8)	3 (4.3)	11 (7.6)	12 (7.2)
No	327 (85.6)	61 (87.1)	123 (84.8)	143 (85.6)
Don’t know	29 (7.6)	6 (8.6)	11 (7.6)	12 (7.2)
**Which other conditions were suspected or investigated before a diagnosis of ILD was confirmed? *n* (%)**	***n* = 26**	***n* = 3**	***n* = 11**	***n* = 12**
Other interstitial lung disease	7 (26.9)	0 (0)	4 (36.4)	3 (25.0)
Asthma	6 (23.1)	2 (66.7)	3 (27.3)	1 (8.3)
COPD	4 (15.4)	1 (33.3)	2 (18.2)	1 (8.3)
Angina	2 (7.7)	1 (33.3)	1 (9.1)	0 (0)
Anxiety	2 (7.7)	1 (33.3)	0 (0)	1 (8.3)
Bronchitis	2 (7.7)	1 (33.3)	0 (0)	1 (8.3)
Congestive heart failure	2 (7.7)	1 (33.3)	0 (0)	1 (8.3)
Pneumonia	2 (7.7)	0 (0)	1 (9.1)	1 (8.3)
Acute bronchitis	1 (3.8)	0 (0)	0 (0)	1 (8.3)
Bronchiectasis	1 (3.8)	0 (0)	1 (9.1)	0 (0)
Emphysema	1 (3.8)	1 (33.3)	0 (0)	0 (0)
Gastroesophageal reflux disease	1 (3.8)	1 (33.3)	0 (0)	0 (0)
Pulmonary hypertension	1 (3.8)	0 (0)	0 (0)	1 (8.3)
Tuberculosis	1 (3.8)	0 (0)	0 (0)	1 (8.3)
Don’t know	1 (3.8)	0 (0)	0 (0)	1 (8.3)

COPD, chronic obstructive pulmonary disease; CTD, connective tissue disease; HCP, healthcare professional; ILD, interstitial lung disease; IPF, idiopathic pulmonary fibrosis.

### 3.5 Symptom burden

**FIGURE 2:**
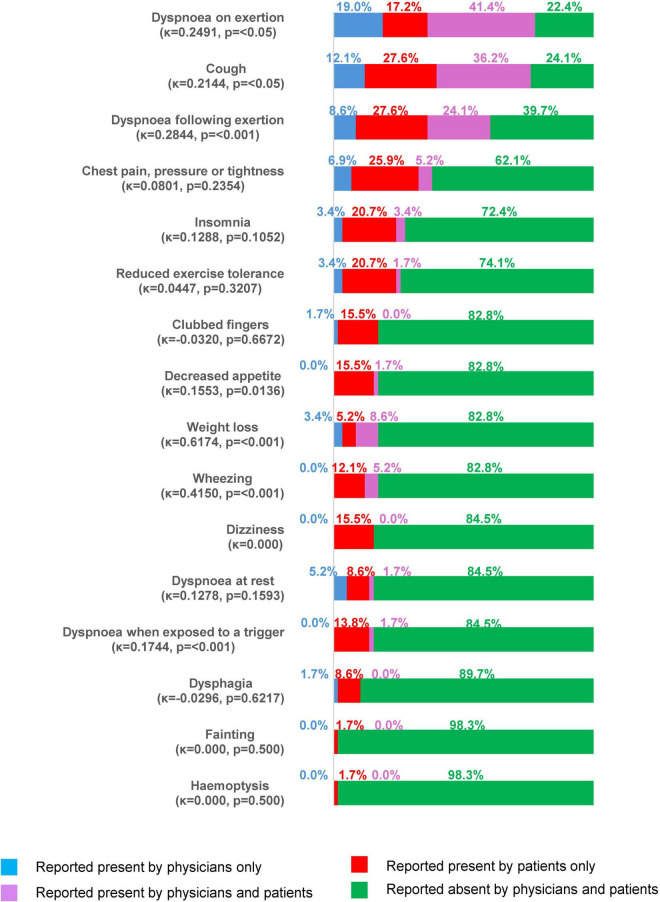
Symptoms reported by physicians or patients at the time of the survey (all patients with both physician- and patient-reported data, *N* = 44).

At the time of the survey, most patients had some symptoms of ILD. As stated by physicans on the PRF, 55/70 patients with IPF (78.6%), 101/145 patients with non-CTD-ILDs (69.7%) and 89/167 patients with CTD-ILDs (53.3%) reported ongoing symptoms in the last 4 weeks, most commonly dyspnoea on exertion (67.4% of all patients with symptoms), cough (57.1%) and dyspnoea following exertion (20.0%), with similar proportions across each type of ILD (Table 7). Other physician-reported signs and symptoms included Velcro crackles (19.2%), chest pressure/tightness (18.0%) and dyspnoea at rest (10.6%). Dyspnoea on exertion was classified as severe by physicians in 10/41 patients with this symptom in the context of IPF, 5/59 patients with non-CTD-ILDs and 6/65 patients with CTD-ILDs (Supplementary Table 2). Based on PSC forms completed by patients (*n* = 58), cough (63.8%), breathlessness on exertion (58.6%) and following exertion (51.7%), and chest pain/tightness (31.0%) were the most common symptoms at the time of the survey (Table 7). Fair alignment was noted for no symptoms being experienced in the 4 weeks prior to the survey date (κ = 0.2529, *p* = <0.05): No symptoms were reported by both physicians and patients in 5.2% of cases, while physicians reported symptoms for 13.8% of patients who did not self-report symptoms, and 5.2% of patients self-reported symptoms when their physician did not. Both physicians and patients reported symptoms in 75.9% of cases. Figure 2 shows the alignment between physicians and patients for the presence/absence of IPF/non-IPF ILD symptoms in the 4 weeks prior to the survey date. Alignment ranged from poor (dysphagia, κ = –0.0296, *p* = 0.6217) to substantial (weight loss, κ = 0.6174, *p* = <0.001). Physicians under-reported symptoms (i.e., a patient-reported symptom was absent from the physician report) in 5.2%–27.6% of patients.

**TABLE 7:** Signs and symptoms reported in the last 4 weeks at the time of the survey.

	All patients	IPF	Non-CTD-ILDs	CTD-ILDs
**Physician-reported patients with symptoms at the time of the survey**	***n* = 382**	***n* = 70**	***n* = 145**	***n* = 167**
Yes	245 (64.1)	55 (78.6)	101 (69.7)	89 (53.3)
No	137 (35.9)	15 (21.4)	44 (30.3)	78 (46.7)
**Physician-reported signs and symptoms**	***n* = 245**	***n* = 55**	***n* = 101**	***n* = 89**
Dyspnoea on exertion	165 (67.4)	41 (74.6)	59 (58.4)	65 (73.0)
Cough	140 (57.1)	31 (56.4)	59 (58.4)	50 (56.2)
Dyspnoea following exertion	49 (20.0)	10 (18.2)	20 (19.8)	19 (21.4)
Velcro crackles	47 (19.2)	8 (14.6)	21 (20.8)	18 (20.2)
Chest pressure/tightness	44 (18.0)	12 (21.8)	20 (19.8)	12 (13.5)
Dyspnoea at rest	26 (10.6)	7 (12.7)	12.9 (13)	6 (6.7)
Fatigue	25 (10.2)	6 (10.9)	8 (7.9)	11 (12.4)
Clubbed fingers	17 (6.9)	7 (12.7)	7 (6.9)	3 (3.4)
Weight loss	16 (6.5)	2 (3.6)	2 (2.0)	12 (13.5)
Reduced exercise tolerance	15 (6.1)	6 (10.9)	4 (4.0)	5 (5.6)
Decreased appetite	9 (3.7)	2 (3.6)	5 (5.0)	2 (2.3)
Insomnia	8 (3.3)	1 (1.8)	3 (3.0)	4 (4.5)
Wheezing	8 (3.3)	2 (3.6)	3 (3.0)	3 (3.4)
Chest pain	7 (2.9)	0 (0)	4 (4.0)	3 (3.4)
No symptoms	6 (2.5)	1 (1.8)	3 (3.0)	2 (2.3)
Tachycardia	5 (2.0)	1 (1.8)	3 (3.0)	1 (1.1)
Dysphagia	3 (1.2)	0 (0)	1 (1.0)	2 (2.3)
Haemoptysis	1 (0.4)	0 (0)	1 (1.0)	0 (0)
Dyspnoea when exposed to trigger	1 (0.4)	1 (1.8)	0 (0)	0 (0)
Don’t know	1 (0.4)	0 (0)	0 (0)	1 (1.1)
**Patient-reported symptoms at time of diagnosis**	***n* = 58**	***n* = 6**	***n* = 29**	***n* = 23**
Yes	52 (89.7)	6 (100)	27 (93.1)	19 (82.6)
No	6 (10.3)	0 (0)	2 (6.9)	4 (17.4)
**Patient-reported symptoms**	***n* = 52**	***n* = 6**	***n* = 27**	***n* = 19**
Cough	37 (71.2)	5 (83.3)	18 (66.7)	14 (73.7)
Breathlessness on exertion	34 (65.4)	4 (66.7)	16 (59.3)	14 (73.7)
Breathlessness following exertion	30 (57.7)	5 (83.3)	13 (48.2)	12 (63.2)
Chest pain/tightness	18 (34.6)	2 (33.3)	6 (22.2)	10 (52.6)
Insomnia	14 (26.9)	0 (0.0)	6 (22.2)	8 (42.1)
Reduced exercise tolerance	13 (25.0)	1 (16.7)	2 (7.4)	10 (52.6)
Decreased appetite	10 (19.2)	0 (0.0)	4 (14.8)	6 (31.6)
Wheezing	10 (19.2)	2 (33.3)	3 (11.1)	5 (26.3)
Swollen/rounded fingertips	9 (17.3)	1 (16.7)	2 (7.4)	6 (31.6)
Dizziness	9 (17.3)	1 (16.7)	3 (11.1)	5 (26.3)
Breathlessness when exposed to a trigger	9 (17.3)	2 (33.3)	2 (7.4)	5 (26.3)
Weight loss	8 (15.4)	0 (0.0)	3 (11.1)	5 (26.3)
Breathlessness at rest	6 (11.5)	1 (16.7)	3 (11.1)	2 (10.5)
Trouble swallowing	5 (9.6)	0 (0.0)	2 (7.4)	3 (15.8)
Coughing up blood	1 (1.9)	0 (0.0)	1 (3.7)	0 (0.0)
Fainting	1 (1.9)	0 (0.0)	1 (3.7)	0 (0.0)
Other	1 (1.9)	0 (0.0)	1 (3.7)	0 (0.0)

CTD, connective tissue disease; HCP, healthcare professional; ILD, interstitial lung disease; IPF, idiopathic pulmonary fibrosis.

### 3.6 Health-related quality of life

Among 65 patients who completed the WPAI-SHP, mean activity impairment was 41.8% (Table 8). Mean impairment while working (23.3%) and overall work impairment (19.1%) were lower, whereas mean work time missed was reported at just 1% (although data were only available for 16–18 patients for these parts of the questionnaire). No notable differences were observed between types of ILD, although patient numbers were low.

**TABLE 8:** Patient-reported Work Productivity and Activity Impairment.

	All patients
Mean work time missed due to problem,	*n* = 17
(SD)	1 (4.04)
Impairment while working due to problem,	*n* = 18
(SD)	23.3 (28.28)
Overall work impairment due to problem,	*n* = 16
(SD)	19.1 (21.93)
Mean activity impairment due to problem,	*n* = 65
(SD)	41.8 (33.95)

SD, standard deviation.

**FIGURE 3:**
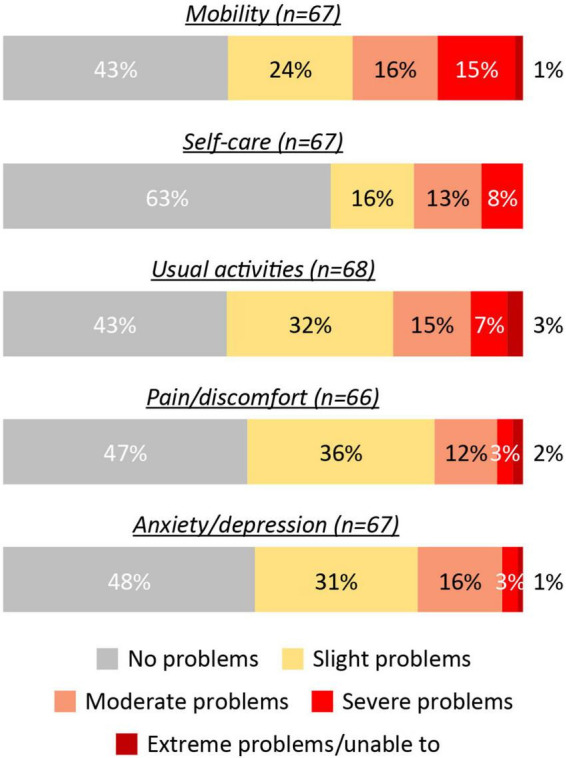
Proportions of patients (all populations) reporting impairment in the EQ5D utility. EQ, EuroQol.

Among 66–68 patients completing different parts of the EQ5D utility, more than half of patients reported problems with mobility (56.7%), usual activities (57.4%), pain/discomfort (57.0%), or anxiety/depression (52.8%), while 37.3% reported problems with self care. These problems mostly ranged from slight to severe, though in a small number of cases (0–3%) the problems were rated as extreme (i.e., the patient felt unable to carry out any relevant activities) (Figure 3).

**FIGURE 4:**
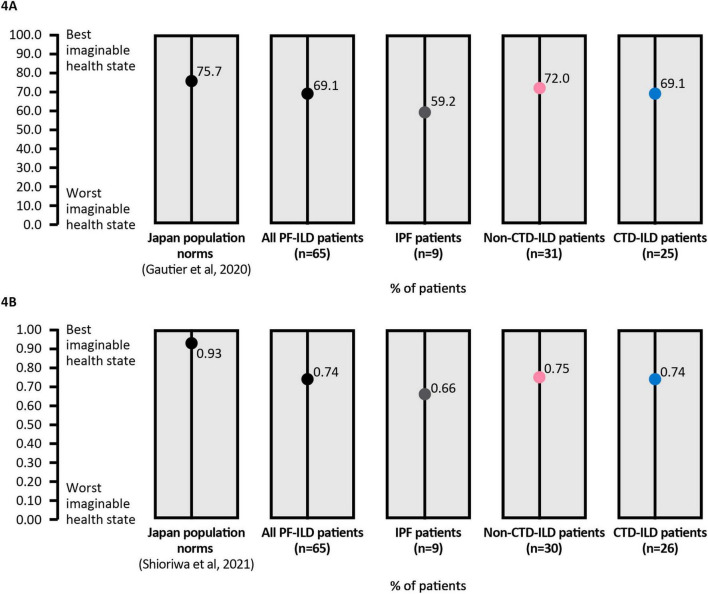
**(A)** EQ5D-5L utility score and **(B)** EQ5D visual analogue scale, in all patients and in subgroups of ILD. Lower scores indicate worse health state. CTD, connective tissue disease; EQ, EuroQoL; ILD, interstitial lung disease; IPF, idiopathic pulmonary fibrosis; PF pulmonary fibrosis.

For the EQ5D-5L score, data were available for 65 patients (9 IPF, 30 non-CTD-ILDs and 26 CTD-ILDs). The mean EQ5D-5L score was 0.74 for all patients (IPF 0.66, non-CTD-ILDs 0.75, CTD-ILDs 0.74), compared with a mean score of 0.93 for the overall Japanese population, indicating a poorer HRQoL for patients in the study. Similarly, for the EQ5D visual analog scale, patient-reported scores were lower (69.1 for all patients) compared with the overall Japanese population (75.7) (Figure 4).

**FIGURE 5:**
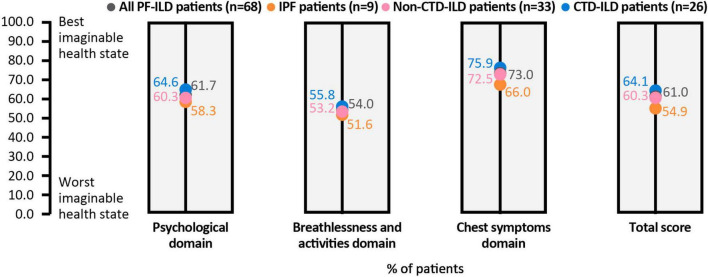
KBILD score, overall and in each domain. All patients and subgroups of ILD are shown as data points within each column. Lower scores indicate worse health state. CTD, connective tissue disease; EQ, ILD, interstitial lung disease; IPF, idiopathic pulmonary fibrosis; KBILD, The King’s Brief Interstitial Lung Disease questionnaire; PF, pulmonary fibrosis.

For the KBILD score, patients reported the impact of their disease on HRQoL, with an overall score for all patients of 61.0/100 (100 equating to best health state and 0 to worst health state) and the highest burden in the breathlessness and activities domain (54.0/100) (Figure 5). Shortness of breath when climbing stairs or walking up an incline was reported by 43% of patients as occurring “every time” or “most times,” whereas 32% of patients reported that they had avoided doing things in the last 2 weeks that made them short of breath “most times” or “a lot of the time.”

## 4 Discussion

The patient journey in PPF is less well understood than that in IPF. To gain greater understanding of the patient journey and burden of PPF in Japan, we have analyzed data from the Adelphi DSP from patients with IPF/PPF in Japan. Considering time from first symptoms to diagnosis, all patients with IPF/PPF experienced a delay in their diagnosis, with those with IPF experiencing the longest mean delay from the onset of symptoms (21.2 months). This may be because patients with PPF due to underlying disease may already be having routine medical examinations when, or soon after, symptoms appear, whereas those with IPF may not have sought treatment until later in their disease. More than half of patients had been referred to their treating physician by another healthcare provider, usually a primary care physician. Primary care physicians may not have the facility to conduct thorough respiratory examinations or 6-minute walk tests to help identify patients with IPF/PPF, and differences in the implementation of the referral process between physicians may have impacted the findings in this study. To improve management of rare diseases such as IPF and PPF, it is important to raise awareness among primary care physicians, and also to establish a referral path to facilitate access to ILD specialists.

The longest delay was between symptom onset and first consultation relating to those symptoms, and was a mean of 14.1 months for those with IPF, 8.0 months for non-CTD-ILDs and 10.7 months for CTD-ILDs. This suggests that patients with IPF may not be seeking help until their condition has worsened significantly. It is important to ensure patients can access information enabling them to identify possible symptoms of ILD. The shorter delay in patients with other forms of ILD may be because they are already receiving care for another condition and therefore physicians are aware of the possibility of ILD. Nevertheless, these patients did not have their first consultation for respiratory symptoms until 8–10 months after symptom onset, so even though they are seeing a healthcare professional, they may not be bringing up any issues around respiratory symptoms and functional capacity, particularly if they are elderly and believe their symptoms to be an inevitable consequence of age. In a patient advisory board (see Supplementary materials), it was suggested that patients may not be aware that certain symptoms are associated with respiratory disease and hence may not bring them up; as a result, primary care physicians may not be aware of the implications of specific symptoms and may not be testing for them. However, it was also indicated that physicians may keep patients with symptoms under observation, rather than treating the symptoms. Following the initial consultation for respiratory symptoms, diagnosis of ILD occurred within 5–7 months.

Despite the delay in diagnosis, fewer than 10% of patients, even those with IPF, were reported as receiving a misdiagnosis prior to their diagnosis of ILD. This is in contrast to previous reports in patients with IPF (6–10) where up to half of patients were reported to be misdiagnosed, although these were not from Japan.

Poor-to-moderate alignment was noted between physician- and patient-reported presence/absence of symptoms (excluding weight loss) in the 4 weeks prior to survey date, with physicians often under-reporting presence. This suggests that patients may not have fully informed physicians of their symptoms. Since patients may not understand the relationship between the disease and symptoms, it is important for physicians to understand patients’ symptoms and problems in daily life, in addition to clinical findings. Previous studies in inflammatory arthritis indicate that factors important to patients may not be adequately addressed even by patient-reported outcome measures (21, 22). As such, improved communication between physicians and patients could ensure that management of disease is targeted toward those areas where patients will feel the most benefit. Due to the relatively small numbers of patients with completed PSCs, it is not possible to compare perception of symptoms between patients and physicians in different types of ILD.

HRQoL was impaired for many patients with pulmonary fibrosis, although up to half of patients reported no impairment, with a consistent pattern across different instruments including WPAI-SHP, EQ5D and KBILD. The impairment of work was less than that of overall activity, which most likely reflects the fact that this population was mostly over the general retirement age in Japan. Compared with the Japanese general population, mean HRQoL was consistently lower in patients in this study, and comparable with HRQoL values previously observed in patients with ILD and in patients with chronic pain (23). HRQoL measures are affected by many systemic influences and therefore may reflect factors other than ILD. For KBILD – an instrument designed specifically for ILD and how frequently ILD symptoms impact daily life – an overall mean burden score of 61.0 and a breathlessness mean domain score of 54.0 demonstrates the severe impact in many patients.

This analysis has limitations, notably the small numbers of patients in some categories. In addition, the time of symptom onset is based on patients’ recall, so the length of delay in diagnosis is not clearly defined. Some parameters, such as the time between diagnosis and ILD progression, were not recorded, and it is not known if differences in HRQoL correlated with differences in pulmonary function and/or duration of disease in different groups. Comparison of HRQoL with the general population was not adjusted for age. These issues limit the conclusions that can be drawn. Nevertheless, this analysis of data from the real-world DSP highlights areas of concern for patients and differences in perception of disease burden between patients and HCPs. These findings add to our understanding of the patient journey and burden of PPF in Japan, and will help guide management that meets the needs of these patients in the future.

## Data Availability

All data, i.e. methodology, materials, data and data analysis, that support the findings of this survey are the intellectual property of Adelphi Real World. The data that support the findings of this study are available from Adelphi Real World, but restrictions apply to the availability of these data, which were used under license for the current study, and so are not publicly available. Requests to access these datasets should be directed to the corresponding author and with permission of Adelphi Real World.
