# Supplementary material for: The patient journey and burden of disease in progressive pulmonary fibrosis in Japan: a cross-sectional survey
Source: Front Med (Lausanne). 2025 Apr 11;12:1526530. doi: 10.3389/fmed.2025.1526530 (PMC12023007; doi:10.3389/fmed.2025.1526530)
Supplement: Supplementary file 1 [file Table_1.docx]

Supplementary Material

# Supplementary Tables

**Supplementary table S1.** PPF-ILD DSP sample size

|  |  | **Physician specialty** | |
| --- | --- | --- | --- |
|  | **Total** | **Pulmonologists** | **Rheumatologists** |
| Physician surveys | 63 | 43 | 20 |
| Patient record forms | 382 | 260 | 122 |
| Patient self-report form | 68 | 49 | 19 |

DSP, Disease Specific Programme; ILD, interstitial lung disease; IPF, idiopathic pulmonary fibrosis; PPF, progressive pulmonary fibrosis.

**Supplementary table S2.** Severity of symptoms in the previous 4 weeks (physician-reported)

|  | **All patients** | **IPF** | **Non-CTD-ILDs** | **CTD-ILDs** |
| --- | --- | --- | --- | --- |
| Chest pain | **7** | **0** | **4** | **3** |
| Mild | 5 | 0 | 3 | 2 |
| Moderate | 2 | 0 | 1 | 1 |
| Severe | 0 | 0 | 0 | 0 |
| Chest pressure/tightness | **44** | **12** | **20** | **12** |
| Mild | 15 | 3 | 7 | 5 |
| Moderate | 23 | 6 | 12 | 5 |
| Severe | 6 | 3 | 1 | 2 |
| Clubbed fingers | **17** | **7** | **7** | **3** |
| Mild | 11 | 4 | 6 | 1 |
| Moderate | 6 | 3 | 1 | 2 |
| Severe | 0 | 0 | 0 | 0 |
| Cough | **140** | **31** | **59** | **50** |
| Mild | 78 | 15 | 32 | 31 |
| Moderate | 57 | 16 | 23 | 18 |
| Severe | 5 | 0 | 4 | 1 |
| Decreased appetite | **9** | **2** | **5** | **2** |
| Mild | 6 | 1 | 3 | 2 |
| Moderate | 2 | 0 | 2 | 0 |
| Severe | 1 | 1 | 0 | 0 |
| Dyspnea at rest | **26** | **7** | **13** | **6** |
| Mild | 3 | 0 | 2 | 1 |
| Moderate | 16 | 2 | 9 | 5 |
| Severe | 7 | 5 | 2 | 0 |
| Dyspnea on exertion | **165** | **41** | **59** | **65** |
| Mild | 65 | 11 | 30 | 24 |
| Moderate | 79 | 20 | 24 | 35 |
| Severe | 21 | 10 | 5 | 6 |
| Dyspnea following exertion | **49** | **10** | **20** | **19** |
| Mild | 15 | 1 | 11 | 3 |
| Moderate | 24 | 5 | 7 | 12 |
| Severe | 10 | 4 | 12 | 4 |
| Dyspnea when exposed to a trigger | **1** | **1** | **0** | **0** |
| Mild | 1 | 1 | 0 | 0 |
| Moderate | 0 | 0 | 0 | 0 |
| Severe | 0 | 0 | 0 | 0 |
| Dysphagia | **3** | **0** | **1** | **2** |
| Mild | 3 | 0 | 1 | 1 |
| Moderate | 0 | 0 | 0 | 0 |
| Severe | 0 | 0 | 0 | 0 |
| Fatigue | **25** | **6** | **8** | **11** |
| Mild | 11 | 3 | 4 | 4 |
| Moderate | 11 | 1 | 3 | 7 |
| Severe | 3 | 2 | 1 | 0 |
| Haemoptysis/hemoptysis | **1** | **0** | **1** | **0** |
| Mild | 1 | 0 | 1 | 0 |
| Moderate | 0 | 0 | 0 | 0 |
| Severe | 0 | 0 | 0 | 0 |
| Insomnia | **8** | **1** | **3** | **4** |
| Mild | 7 | 0 | 1 | 1 |
| Moderate | 1 | 1 | 0 | 0 |
| Severe | 0 | 1 | 0 | 0 |
| Reduced exercise tolerance | **15** | **6** | **4** | **5** |
| Mild | 2 | 1 | 1 | 0 |
| Moderate | 9 | 4 | 1 | 4 |
| Severe | 4 | 1 | 2 | 1 |
| Tachycardia | **5** | **1** | **3** | **1** |
| Mild | 2 | 0 | 1 | 1 |
| Moderate | 3 | 1 | 2 | 0 |
| Severe | 0 | 0 | 0 | 0 |
| Velcro crackles | **47** | **8** | **21** | **18** |
| Mild | 22 | 4 | 12 | 6 |
| Moderate | 21 | 3 | 7 | 11 |
| Severe | 4 | 1 | 2 | 1 |
| Wheezing | **8** | **2** | **3** | **3** |
| Mild | 2 | 0 | 1 | 1 |
| Moderate | 5 | 1 | 1 | 2 |
| Severe | 1 | 0 | 1 | 0 |
| Weight loss | **16** | **2** | **2** | **12** |
| Mild | 10 | 1 | 1 | 8 |
| Moderate | 6 | 1 | 1 | 4 |
| Severe | 0 | 0 | 0 | 0 |
