# Supplementary material for: The patient journey and burden of disease in progressive pulmonary fibrosis in Japan: a cross-sectional survey
Source: Front Med (Lausanne). 2025 Apr 11;12:1526530. doi: 10.3389/fmed.2025.1526530 (PMC12023007; doi:10.3389/fmed.2025.1526530)
Supplement: Supplementary file 2 [file Data_Sheet_1.docx]

Supplementary Material

# J-BREATH Advisory Board Meeting | Minutes

**14:00-15:00 J-BREATH Advisory Board Meeting on Friday March 22, 2024.**

**Group representatives: J**

**Patients: S**

1. **Manifestations**


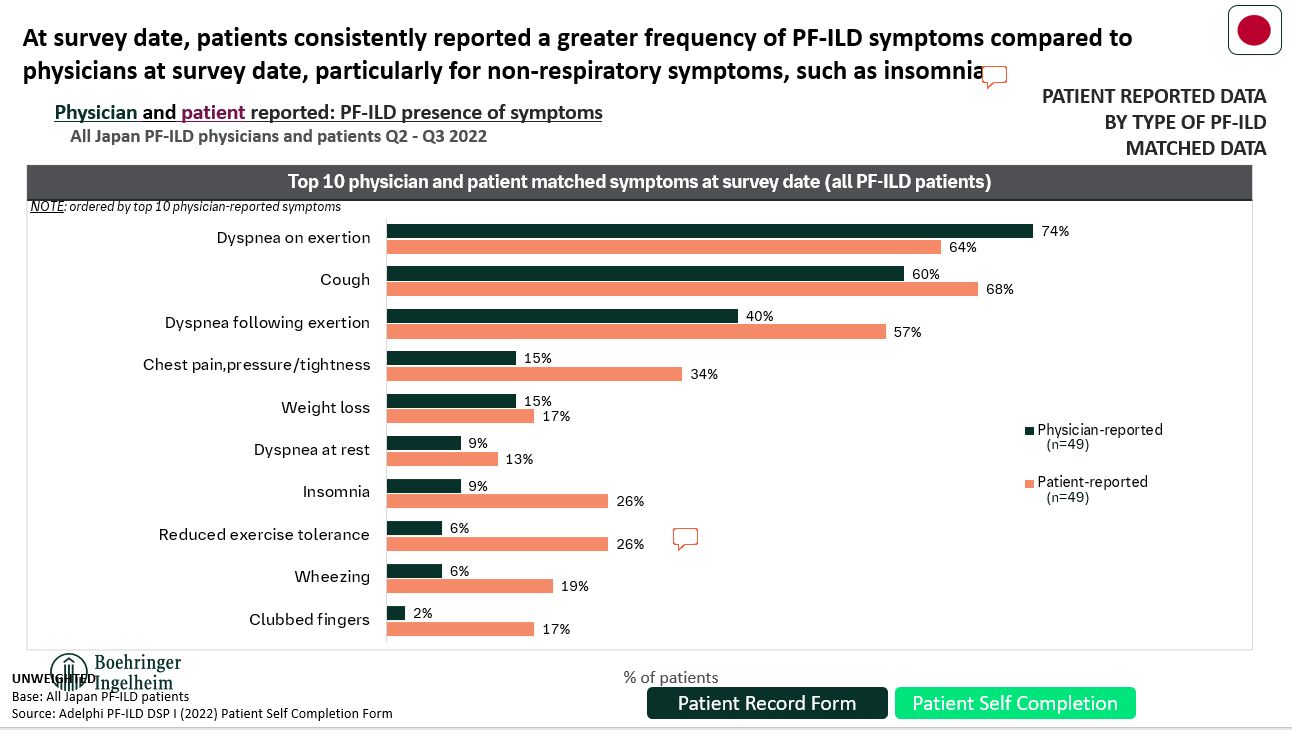


**UNWEIGHTED**

① Different symptoms are generally perceived by physicians and patients, especially in non-respiratory symptoms such as insomnia, but where does this communication gap come from? (problems on both physician and patient sides)
J: Since these symptoms does not appear in the examinations, it is difficult to the physician to know, because it is a patient feelings. I’m not sure that the association between insomnia and disease.
S:I have insomnia but don't want to take medicine well or talk to the doctor. Since the sleep was short originally, I thought that it was a constitution. I thought that insomnia was not related to the diseases, so I had no idea of consulting a doctor. I was able to tell if the doctor asked me

② What do you think can resolve the gap?
S: If I get sick, I’m looking at the net, but patients don’t complain about the symptoms until they know from their doctors that there are such symptoms. Insomnia is common among patients. Because I don't know that insomnia = respiratory disease. Doctors don't hear if they sleep. I don't talk to physicians about symptoms in detail. I'm used to not being able to sleep.

1. **Problematic presentation**


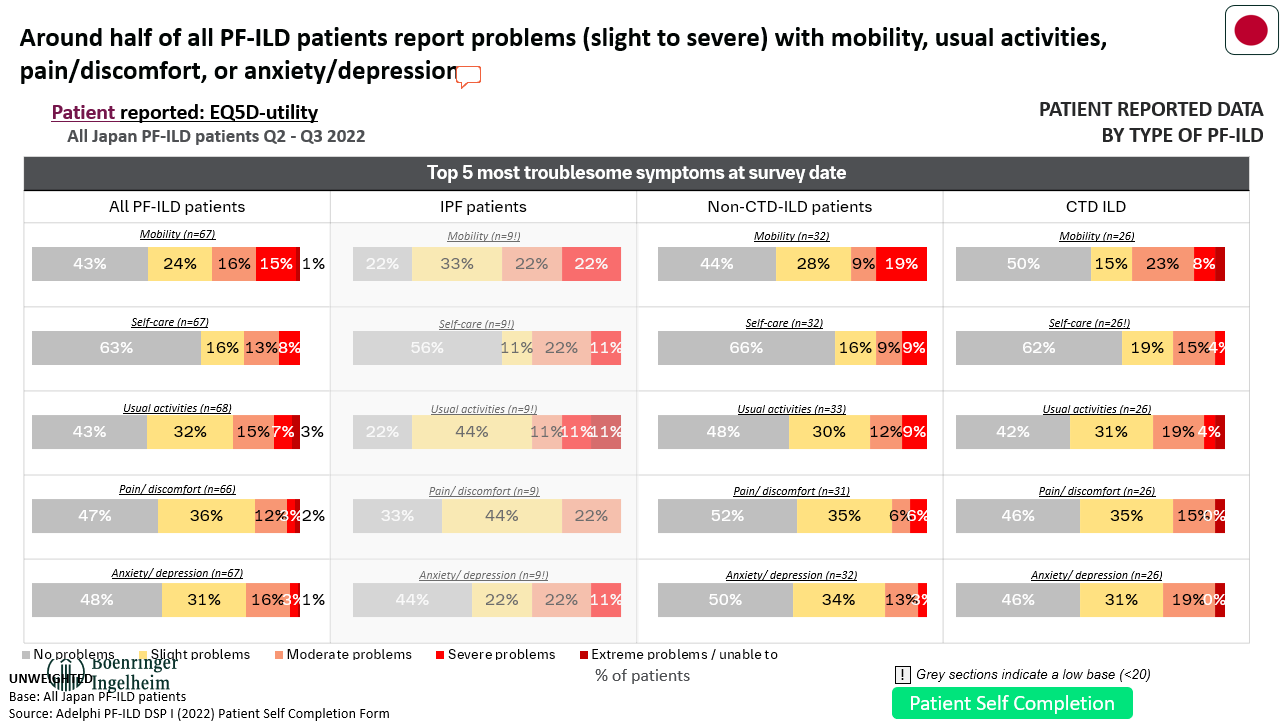


**UNWEIGHTED**

① What are the specific restrictions?

S: They usually live. If I notice, breathe through the mouth, and I do not exercise vigorously. I try my best to go up the short stairs. Drug side effects may limit behavior. Barrier-free may be the best way to go, but I should exercise a little in my daily life, so I do some shopping and walking.

② Do patients who do not feel much of the problem really think they do not have problems in their daily lives? Why do they take behavioral restrictions as a matter of course?
S: I think it's better to go outside and have fun as much as possible. I sometimes canceled my journey because of drug side effects. Sometime, I wanted to stop taking medicine because drug side effects limit activities.
J: Depending on severity. Though in the mild disease, the action is usually carried out without change, it is very difficult for some people to stand on exertion and tray, when oxygen is required. Some people are able to move out of oxygen if they sit, but if they move a little, they cannot move if they do not have a large amount of oxygen.

1. **Work**


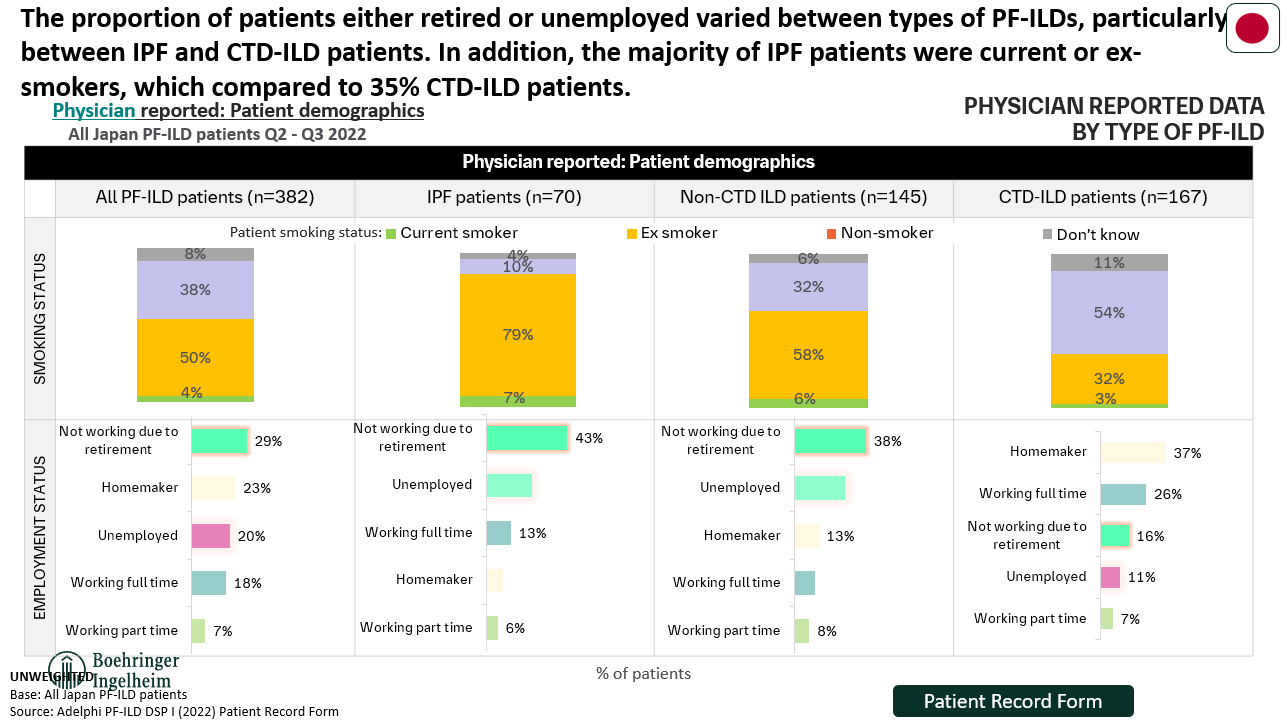


**UNWEIGHTED**

① What are the difficulties in getting to work and working while living with interstitial lung disease? (Retired and unemployed patients who want to work but cannot do so)
J: There is a tendency for more patients to be bothered by talking to a company. After that it is judgement whether they can continue or not. When a patient is diagnosed as having a progressive disease and disclose to their company, they consider the burden on the companions that makes it difficult to do work. People who aim to be a career do not want to speak to the company, but they should change the career plan that they were drawing because suddenly falling down causes trouble to the company.

② What kind of support and public systems, including work and housework, should be available? (Expectations: ILD also affects the social life of the patient in terms of employment)

J: The company and society should understand that while working, they may be able to work, but they may become ill. There is also an economic burden. The economic burden between working and not working is different. It may be worried whether it is better to devote to medical treatment. It's good to have a place where they can consult with them, because they can be just said that everyone is different. It cannot be done by the patient group to refer a job referral, and we can only say in general. Support and options are needed. It is also necessary to designate intractable disease.

1. **Disease progression**
   1. **Doctor**


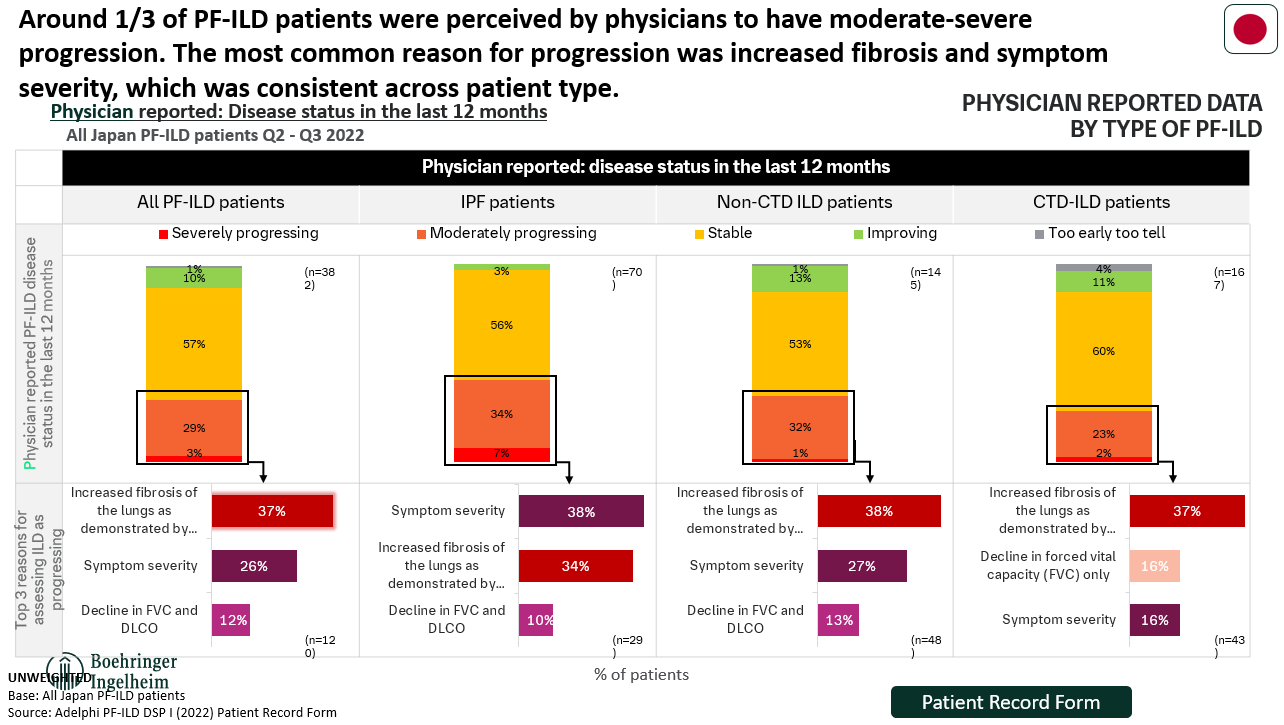


**UNWEIGHTED**

Slide P10

① How to explain the seriousness by the physician on the rationale
S: Lung function was said to be only 70% when diagnosed. One year after he started to take the medicine, I heard about the progress, but it was said that it had not progressed so much, so I thought that I would have taken the medicine. Different hospitals have different support systems. X-rays, blood tests, and vital capacity (6-minute walk) are measured. My physician only passes a data sheet for the test, but I want to the physician to explain it carefully. The values of the blood test can be shown, and some results can be understood that is not very good, but I can’t fully understand since I don’t know the standard values of data.

② Is the physician's explanation highly convincing? If not high, what explanation is necessary?

S: If I go to the doctor's consultation by examining the disease on my own, it would be better not to examine it because the doctor feel bad and he says it is such an armchair theorist. I have to ask him.
J: Interstitial pneumonia has a poor prognosis in some patients, and it is impressive that there is a lot of information that the prognosis is poor. However, because the classification of the disease is difficult, we asked the patients who are easy to touch with the information that the prognosis is bad. They said “It was not good that they don’t know what will happen in the future, so it is important to explain that if it is good or bad. Though there is a difficult side to attend when it is bad, it is important to attend to the patient first. It is also working on rehabilitation”. It is important to explain that if it gets bad, it is not left when it is said that it is bad.

S: Different hospitals handle diseases differently. Some doctors call nurses and family members to give explanations. As my hospital does not respond so far, I think that it is good for hospitals that are responding properly.

③ At what symptoms do you feel the severity?

S: It does not seem to have progressed compared to last year. Blood test results may indicate that the liver value is poor, but the drug is still the cause. Drugs are effective. Shortness of breath does not change particularly.
J: Many patients say that cough is the most difficult. Some patients may say that mint has any way to relieve cough. Though shortness of breath seems to be parallel, there are many complaints of the desire to make cough most, because it is very difficult to have a cough for both eating and speaking.
S:I got rid of all the feathers in my house because the feathers aggravated the symptoms. Everyone is wearing feathered clothes in winter, so it is painful.
